# A novel survival model based on a Ferroptosis-related gene signature for predicting overall survival in bladder cancer

**DOI:** 10.1186/s12885-021-08687-7

**Published:** 2021-08-21

**Authors:** Yingchun Liang, Fangdie Ye, Chenyang Xu, Lujia Zou, Yun Hu, Jimeng Hu, Haowen Jiang

**Affiliations:** 1 Departments of Urology, Huashan Hospital, Fudan University, No. 12 WuLuMuQi Middle Road, Shanghai, 200040 China; 2 Fudan Institute of Urology, Huashan Hospital, Fudan University, Shanghai, China; 3 National Clinical Research Center for Aging and Medicine, Fudan University, Shanghai, China

**Keywords:** Bladder cancer, Ferroptosis, Prognostic model, Gene signature, The Cancer genome atlas (TCGA)

## Abstract

**Background:**

The effective treatment and prognosis prediction of bladder cancer (BLCA) remains a medical problem. Ferroptosis is an iron-dependent form of programmed cell death. Ferroptosis is closely related to tumour occurrence and progression, but the prognostic value of ferroptosis-related genes (FRGs) in BLCA remains to be further clarified. In this study, we identified an FRG signature with potential prognostic value for patients with BLCA.

**Methods:**

The corresponding clinical data and mRNA expression profiles of BLCA patients were downloaded from The Cancer Genome Atlas (TCGA). Univariate Cox regression was used to extract FRGs related to survival time, and a Cox regression model was used to construct a multigene signature. Both principal component analysis (PCA) and single-sample gene set enrichment analysis (ssGSEA) were performed for functional annotation.

**Results:**

Clinical traits were combined with FRGs, and 15 prognosis-related FRGs were identified by Cox regression. High expression of CISD1, GCLM, CRYAB, SLC7A11, TFRC, ACACA, ZEB1, SQLE, FADS2, ABCC1, G6PD and PGD was related to poor survival in BLCA patients. Multivariate Cox regression was used to construct a prognostic model with 7 FRGs that divided patients into two risk groups. Compared with that in the low-risk group, the overall survival (OS) of patients in the high-risk group was significantly lower (*P* < 0.001). In multivariate regression analysis, the risk score was shown to be an independent predictor of OS (HR = 1.772, *P* < 0.01). Receiver operating characteristic (ROC) curve analysis verified the predictive ability of the model. In addition, the two risk groups displayed different immune statuses in ssGSEA and different distributed patterns in PCA.

**Conclusion:**

Our research suggests that a new gene model related to ferroptosis can be applied for the prognosis prediction of BLCA. Targeting FRGs may be a treatment option for BLCA.

**Supplementary Information:**

The online version contains supplementary material available at 10.1186/s12885-021-08687-7.

## Background

Bladder cancer (BLCA) is a global problem and has been reported as the ninth most common tumour in the world [1, 2]. More than 200,000 people died from the disease, and over 549,000 new cases were diagnosed in 2018 [1, 2]. In the past 20 years, the number of BLCA cases has been increasing worldwide. Due to population ageing, environmental pollution and smoking [3], the number of BLCA cases may increase in the future [4, 5]. Although many treatments, including surgery, chemotherapy, and radiotherapy, have been performed on BLCA patients, BLCA has a high risk of progression, metastasis, recurrence and poor prognosis [6, 7]. Therefore, exploring and discovering reliable prognostic biomarkers is important to guide clinical treatment and improve the prognosis of BLCA.

Ferroptosis is an iron-dependent form of programmed cell death driven by the accumulation of lipid peroxides [8, 9], which is different from autophagic cell death or traditional apoptosis or necrosis [8]. In recent years, promoting ferroptosis has become a promising treatment option that causes cancer cell death, especially for malignant tumours that are resistant to conventional treatment [10, 11]. In addition to drugs that cause ferroptosis, many genes have also been identified as regulators or markers of ferroptosis. Ferroptosis regulatory genes such as GPX4 [12], P53 [13], DPP4 [14], HSPB1 [15] and FANCD2 [16] are closely related to tumour occurrence and progression. An increasing number of studies have reported that a variety of tumour cells, including hepatocellular carcinoma cells [17], ovarian cancer cells [18] and adrenocortical carcinoma cells [19], are sensitive to ferroptosis. In addition, the combination of chemotherapeutic drugs and erastin, a ferroptosis inducer, can improve the therapeutic effect on lung cancer cells [20], acute ovarian cancer cells [21], gastric cancer cells [22] and myeloid leukaemia cells [23]. Therefore, ferroptosis may become a potential target for cancer treatment. However, the relationship between the prognosis of BLCA patients and the expression of ferroptosis-related genes (FRGs) has not been studied in detail.

In the present study, the corresponding clinical data and mRNA expression profiles of BLCA patients were downloaded from The Cancer Genome Atlas (TCGA) database. Then, a prognostic multigene signature was constructed based on 7 FRGs to predict the survival of BLCA patients. Overall, our data indicate that FRGs play a key role in the pathogenesis of BLCA and are potential therapeutic targets and prognostic markers for BLCA.

## Materials and methods

### Data collection

The publicly available RNA sequencing (RNA-seq) data and corresponding clinical information of 433 patients with BLCA samples (*n* = 414) and normal bladder samples (*n* = 19) were downloaded from the TCGA website (https://gdc.cancer.gov/). We used the “limma” R package to identify the gene expression profiles in the TCGA BLCA dataset. The data from TCGA are publicly available. Therefore, this study did not need to be approved by the local ethics committee.

### FRG acquisition

Sixty FRGs were acquired from previous research [9, 10, 24, 25] and are presented in Supplementary Table S1.

### Construction of a prognostic-related FRG risk score model

FRG expression was converted to a logarithmic value based on 2 and then associated with clinical features. Univariate Cox regression was used to extract FRGs related to survival time, using *p* < 0.05 as the threshold. The STRING database (version 11.0) was used to generate an interactive network of prognosis-related FRGs [26]. Multivariate Cox regression was used to construct a multigene signature and calculate the risk score for each patient [27, 28]. The risk score of the FRG signature for each patient was calculated according to the following formula: Risk score = (− 0.3722 × Expression of ACSL4) + (− 0.1217× Expression of ALOX5) + (0.1718× Expression of GCLM) + (0.3100× Expression of ACACA) + (0.3463 Expression of ZEB1) + (0.0967 × Expression of FADS2) + (− 0.1884 × Expression of NOX1). The patients were divided into high-risk and low-risk groups based on the median value of the risk score. Furthermore, the K-M survival curve and risk curve were analysed based on survival information and the risk score.

### Independent prognostic analysis

The clinical characteristics and risk scores were compared with survival time and survival status to determine whether the risk score can be used as an independent prognostic factor. The time-dependent receiver operating characteristic (ROC) curve was used to evaluate the accuracy of the model in predicting the prognosis of BLCA, and the “survivalROC” package in R was used to measure the area under the curve (AUC) of risk score, age, sex, stage, T stage, N stage, and M stage.

### Clinical relevance analysis

Box plots were used to analyse whether clinical features were associated with the FRGs in the risk score model.

### Bioinformatics analysis

Principal component analysis (PCA) was performed by using the “scatterplot3d” R package to profile the expression patterns of the grouped samples. Single-sample gene set enrichment analysis (ssGSEA) with the “gsva” R package was utilized to calculate the immune infiltrating score of 16 immune cells and the expression of 13 immune-related pathways [29]. The relevant annotated gene set file is provided in Supplementary Table S2. To present the potential functions of the differentially expressed genes (DEGs) between the low-risk and high-risk groups, the “clusterProfiler” R package was used to perform Gene Ontology (GO) enrichment analysis and Kyoto Encyclopedia of Genes and Genomes (KEGG) pathway [30] enrichment analysis.

### Cell culture and siRNA knockdown

The human BLCA cell line T24 was obtained from the Type Culture Collection at the Chinese Academy of Sciences. The siRNA against ZEB1and FADS2 were purchased from GenePharma (Shanghai, China). T24 cells were cultured in DMEM (Gibco, Grand Island, NY, USA) containing 10% foetal bovine serum at 37 °C with 5% CO_2_. T24 cells were transfected with 20 nmol/L siRNAs using Lipofectamine 2000 RNAiMAX reagent (Invitrogen, Carlsbad, CA, USA).

### Cell proliferation assay

We seeded 2000 T24 cells per well into 96-well culture plates for 5 days in triplicate wells. Cell Counting Kit 8 (Gibco) was used according to the manufacturer’s instructions. Then, 10 μL of CCK-8 reagent was added to each well and incubated for 1–2 h. The optical density (OD) values of each well were determined at 450 nm using a microplate reader.

### Statistical analysis

All statistical analyses were conducted using R software (Version 3.5.3) and SPSS (Version 23.0). The overall survival (OS) between the high-risk and low-risk groups was compared by K-M analysis with the log-rank test. Univariate and multivariate Cox regression analyses were employed to analyse independent predictors of OS. The Mann-Whitney test was used to compare the ssGSEA scores of immune cells or immune-related pathways between the different groups. Unless otherwise specified, a *P*-value < 0.05 was considered statistically significant.

## Results

### Identification of prognostic factors and FRGs in the TCGA-BLCA cohort

**Fig. 1:**
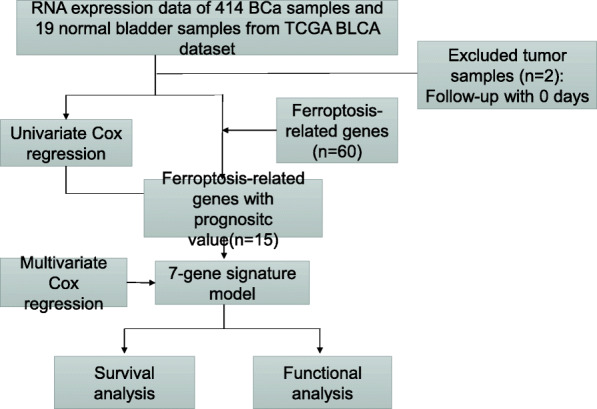
Flowchart of this study

**Fig. 2:**
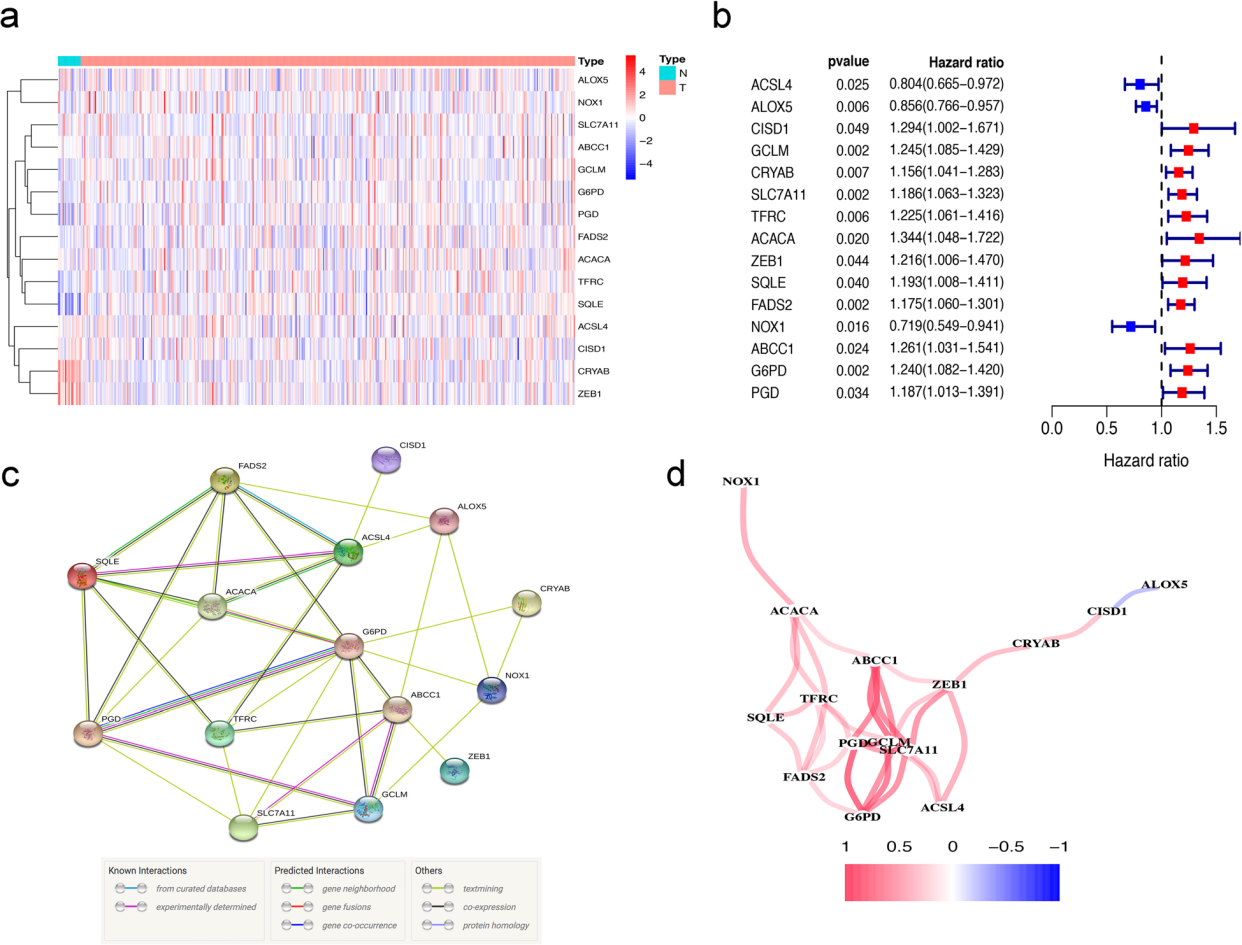
Identification of prognostic factors and FRGs. (**a**) Heatmap for the expression of 15 FRGs in BLCA patient samples. N stands for tumor sample and T stands for normal sample. Up-regulated FRGs are shown in red, while down-regulated FRGs are shown in blue. (**b**) Forest plot presents the results of the univariate Cox regression analysis for the 15 FRGs between gene expression and OS. (**c**) The PPI network shows the interaction between candidate genes. (**d**) The correlation network between candidate FRGs. Different colors represent the correlation coefficients

The flowchart in Fig. 1 is a brief summary of the present study. A total of 412 BLCA patients from the TCGA-BLCA cohort were finally enrolled. Fifteen (15/60, 25%) FRGs were correlated with prognosis in the univariate Cox regression analysis (all FDRs < 0.05, Fig. 2a-b). The results showed that the high expression of CISD1, GCLM, CRYAB, SLC7A11, TFRC, ACACA, ZEB1, SQLE, FADS2, ABCC1, G6PD and PGD was related to poor survival rates in BLCA patients. In contrast, the high expression of ACSL4, ALOX5 and NOX1 was associated with a higher survival rate in BLCA patients (Fig. 2a-b). The protein-protein interaction (PPI) network of the 15 ferroptosis-related proteins indicated that PGD, G6PD and ABCC1 were the hub genes of these FRGs (Fig. 2c). The correlation coefficients between these FRGs are presented in Fig. 2d.

### Construction of a prognostic FRG risk score model

**Fig. 3:**
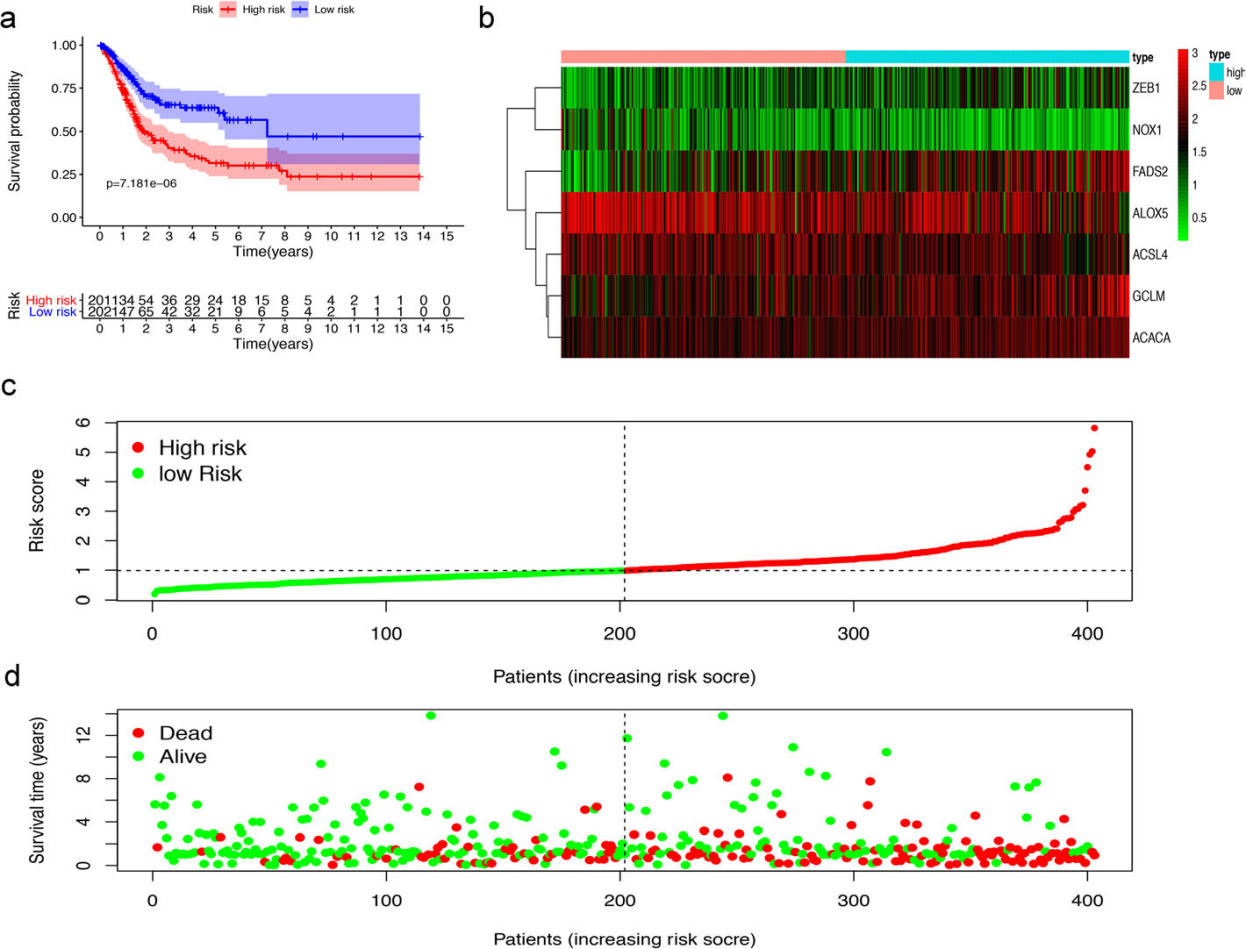
Prognostic evaluation of the 7-FRGs signature in BLCA patients. (**a**) The Kaplan–Meier survival curve shows overall survival of high- and low-risk BLCA patients. (**b**) The heat map of expression profiles of 7-FRGs in high- and low-risk BLCA patients. (**c**, **d**) Distribution of risk score, survival status of patients based on data. (**c**) The low-risk curve is green and the high-risk curve is red. (**d**) Red dots indicate people who are dead and green dots indicate people who still alive

Of the above prognostic-related FRGs, 7 prognostic-related FRGs were screened out through multivariate Cox regression analyses (Table 1). The prognostic model was used to establish and calculate the risk scores of all patients. The patients were divided into a high-risk group (*n* = 201) and a low-risk group (*n* = 202). The survival of the low-risk group was significantly higher than that of the high-risk group (median OS 7.236 years versus 1.907 years, *P* < 0.001, Fig. 3a). The heat map of the expression profiles of the seven FRGs is displayed in Fig. 3b. The risk score and survival time of each patient are shown in Fig. 3c, d. The survival time of patients in the high-risk group was significantly lower than that of the low-risk group. Compared with the low-risk group, the number of deaths increased faster in the high-risk group.

**Table 1:** Regression coefficient of 7 FRGs based on multivariate Cox regression

ID	coef	HR
ACSL4	−0.372215323	0.689205825
ALOX5	−0.121694795	0.885418562
GCLM	0.171781879	1.187418804
ACACA	0.309998606	1.363423213
ZEB1	0.34630541	1.41383435
FADS2	0.096672823	1.101499929
NOX1	−0.188401336	0.828282221

Note. *ACSL4* long-chain Acyl-CoA synthetase 4, *ALOX5* Arachidonate-5-Lipoxygenase, *GCLM* modifier subunit of glutamate-cysteine ligase, *ACACA* Acetyl-CoA carboxylase alpha, *ZEB1* E-box-binding homeobox 1, *FADS2* fatty acid desaturase-2, *NOX1* NADPH oxidase 1

### Independent prognostic analysis and clinical relevance analysis

**Fig. 4:**
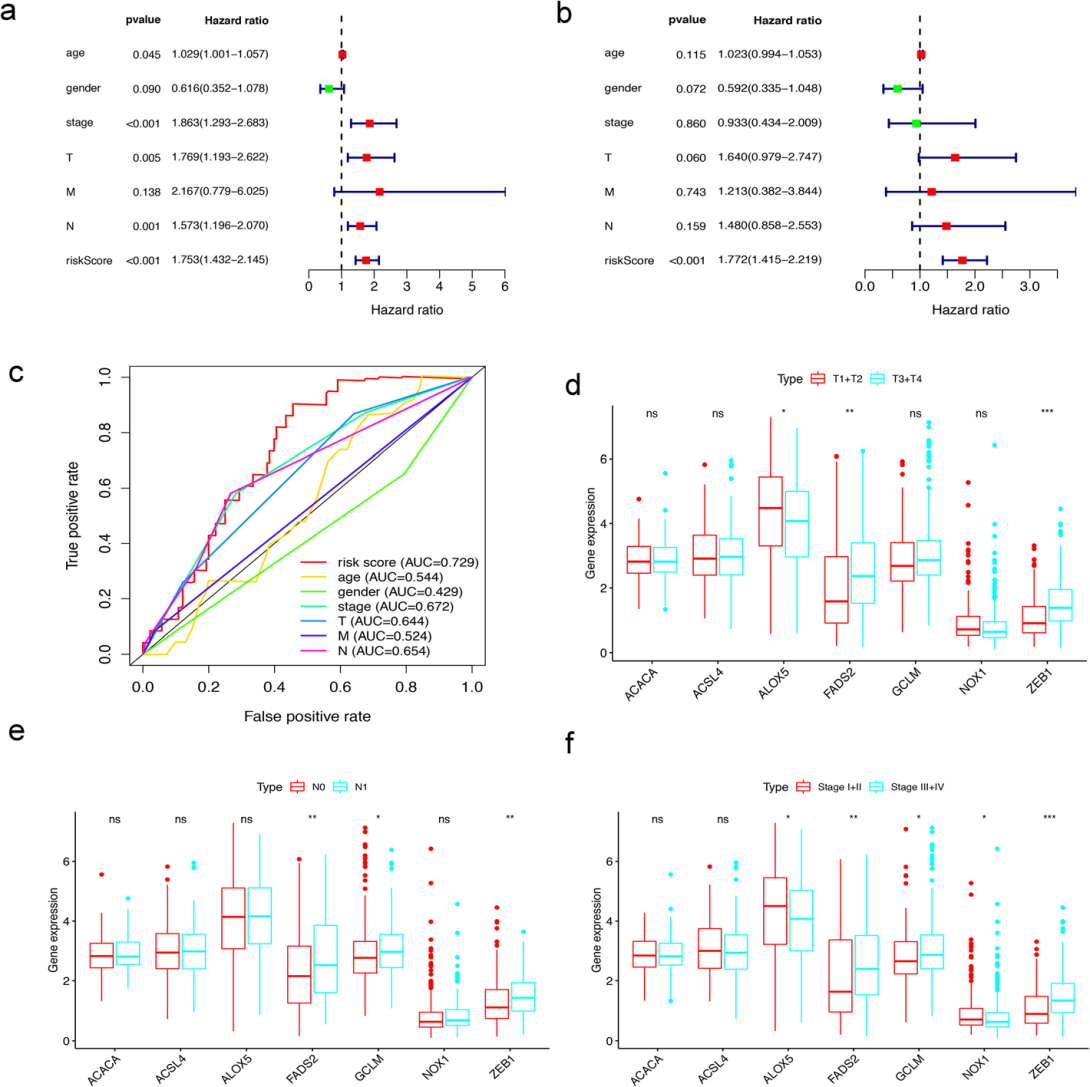
Evaluating the predictive power of 7-FRG signature and clinical relevance analysis. (**a**, **b**) Forest plot show the results of univariate and multivariate Cox regression analysis in the TCGA BLCA cohort. (**c**) ROC curves and AUCs based on the TCGA-BLCA cohort for 5-year OS. (**d**, **e**, **f**) Relationship between FRGs with T stages, N stages and clinical stages. (“ns” means they are not significant; *, *P* < 0.05; **, *P* < 0.01; ***, *P* < 0.001)

Among all the clinical features, univariate Cox regression analysis revealed that age, clinical stage, T stage, N stage, and risk score were related to OS in TCGA-BLCA (*p* < 0.05) (Fig. 4a). Among them, risk score was an independent prognostic factor in multivariate Cox regression for BLCA patients (*p <* 0.05), but age, clinical stage, T stage, and N stage were not (Fig. 4b). Furthermore, we generated ROC curves to represent the prognostic predictive value of these clinical features. The AUC of sex was 0.429, while the AUCs of all other factors, including age, clinical stage, T stage, M stage, N stage and risk score, were over 0.500. Among them, the AUC of the risk score was 0.729 (Fig. 4c). Therefore, the results proved that the risk score calculated by the model can accurately predict the 5-year survival rate of BLCA patients. Box plots were used to analyse the correlation between the FRGs and clinical traits. The results showed that T stage was related to ALOX5, FADS2, and ZEB1; N stage was associated with FADS2, GCLM, and ZEB1; and clinical stage was associated with ALOX5, FADS2, GCLM, NOX1 and ZEB1 (Fig. 4d-f).

### Analysis of the high- and low-risk populations by PCA and ssGSEA

**Fig. 5:**
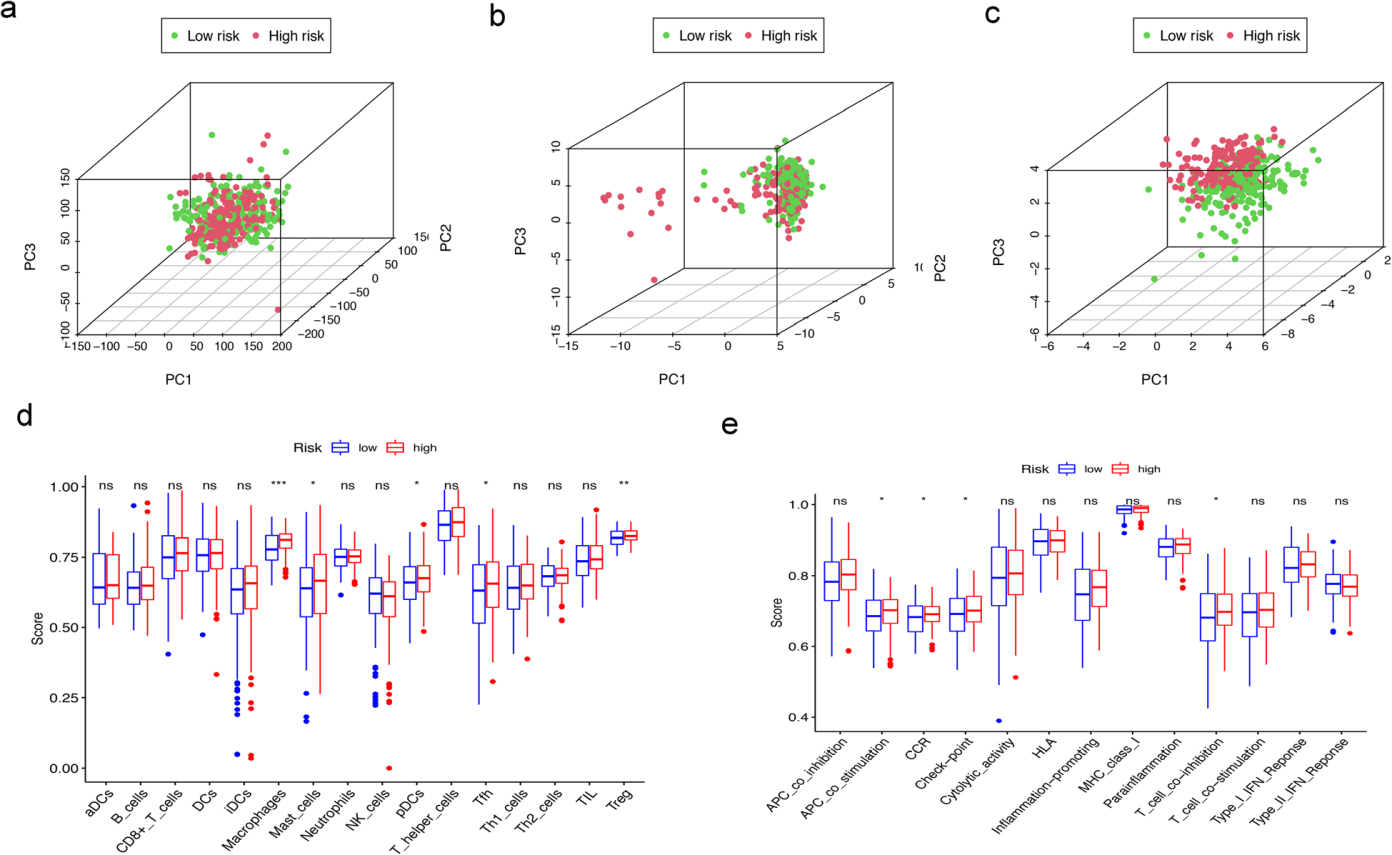
Analysis of the high- and low-risk populations by PCA and ssGSEA in the TCGA-BLCA cohort. (**a**, **b**, **c**) PCA between high- and low-risk groups is conducted based on the whole protein coding gene, FRG, and 7-FRG signature sets. (**d**, **e**) Comparison of the ssGSEA scores of 16 immune cells and 13 immune-related functions between different risk groups in the TCGA cohort. (“ns” means they are not significant; *, *P <* 0.05; **, *P <* 0.01; ***, *P <* 0.001)

We used PCA to detect the distribution patterns of different risk states between the high- and low-risk groups through the whole protein coding gene, FRG and 7-FRG signature sets. In the 7-FRG signature set, the high- and low-risk groups were clearly separated (Fig. 5a). However, the whole protein coding gene and FRG sets could not distinguish the high- and low-risk groups well. In other words, 7 FRGs were used to divide BLCA patients into two groups, indicating that there is a large difference between BLCA patients in the high- and low-risk groups (Fig. 5b-c).

To explore the correlation between immune status and the risk score, we used ssGSEA to quantify the enrichment scores of different immune cell subsets and related pathways. Interestingly, the content and scores of immune cell subsets and related pathways, including Macrophages, Mast_cells, pDCs, Tfh, Treg, APC_co_stimulation, CCR, Check-point and T_cell_co-inhibition in the high-risk group were significantly higher than those in the low-risk group (*P* < 0.05, Fig. 5d-e).

### Functional analyses in the TCGA cohort

**Fig. 6:**
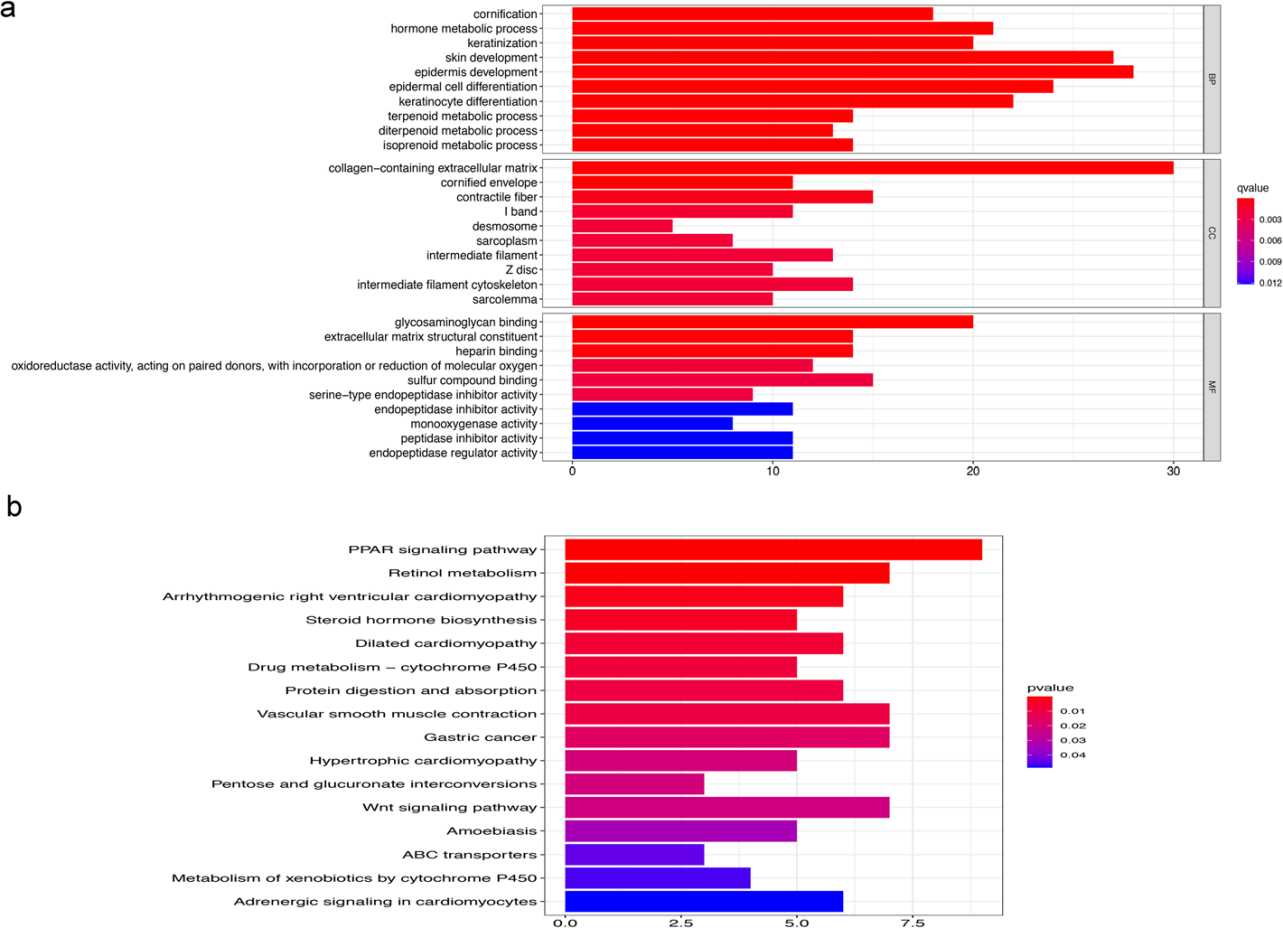
Representative results of GO and KEGG analyses. (**a**) GO terms show the main biological processes represented by the DEGs between the low- and high-risk groups. (**b**) KEGG pathway analyses represent the main signalling pathways for the DEGs between the low- and high-risk groups

To clarify the biological functions and pathways related to the risk score, the DEGs between the high- and low-risk groups were used for GO enrichment and KEGG pathway analysis. The molecular functions and biological processes of these genes were obviously enriched in epidermal cell differentiation, skin development, keratinocyte differentiation, collagen-containing extracellular matrix and glycosaminoglycan binding. KEGG pathway analyses also demonstrated that the PPAR signalling pathway, retinol metabolism and vascular smooth muscle contraction were the most significant pathways in the TCGA cohort (*P* < 0.05, Fig. 6a, b).

### Validation of gene expression and function

**Fig. 7:**
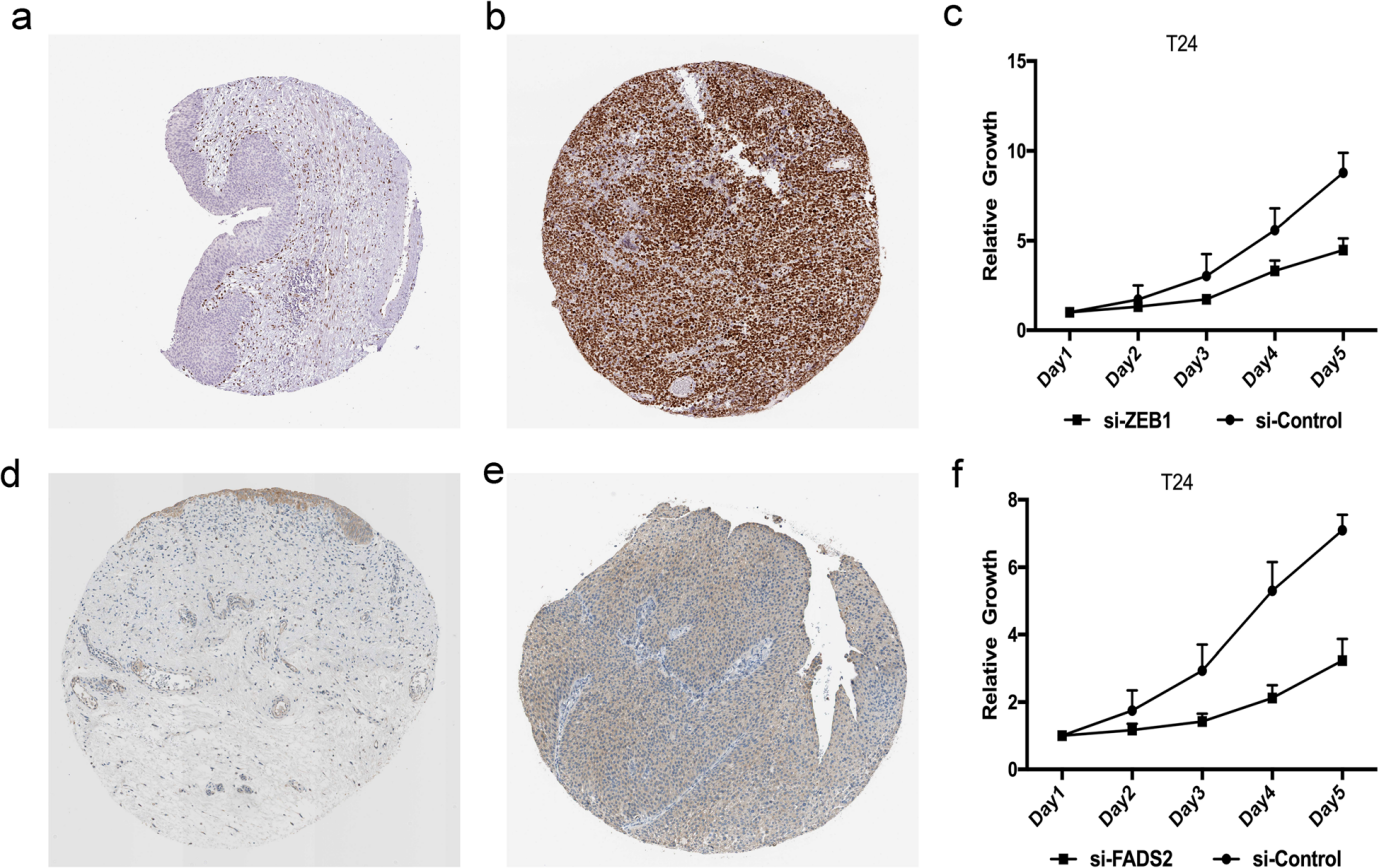
Experimental verification of ZEB1 and FADS2. Immunohistochemical images from the HPA database show ZEB1 protein expression in normal bladder (**a**) and BLCA (**b**) tissues. (**c**) CCK-8 assay results show the relative proliferation of si-control- and si-ZEB1-transfected T24 cells. The data is presented as means ± SD. Immunohistochemical images from the HPA database show FADS2 protein expression in normal bladder (**d**) and BLCA (**e**) tissues. (**f**) CCK-8 assay results show the relative proliferation of si-control- and si-FADS2-transfected T24 cells

Next, we used the ZEB1 and FADS2 genes to verify our model. The Human Protein Atlas database analysis showed that ZEB1 and FADS2 expression was significantly higher in BLCA tissues than in normal bladder tissues (Fig. 7a, b, d, e). CCK-8 proliferation analysis demonstrated that compared with control T24 cells, ZEB1- and FADS2-knockdown T24 cells showed a significant reduction in proliferation (*P <* 0.05, Fig. 7c, f).

## Discussion

The conventional treatment of BLCA is surgery and chemotherapy, but it is not sufficient. The most effective anticancer treatment is to selectively induce cancer cell death. Increasing evidence shows that ferroptosis plays a vital role in tumorigenesis and cancer treatment. However, the distribution of ferroptosis in BLCA has not been confirmed. Based on current conditions, we tried to extract the transcriptome sequencing data of BLCA from the TCGA public database and then systematically analysed the expression of 60 FRGs in BLCA tissues and adjacent nontumorous tissues and their relationship with OS. The results of univariate Cox regression analysis revealed that 15 FRGs were significantly related to the survival time of BLCA patients, which supports our view that FRGs may be related to the regulation of the antitumour process. Then, multivariate Cox regression showed that a new prognostic model integrating 7 FRGs can distinguish patients with poor prognosis from those with good prognosis. This prognostic model performed better than the widely used clinical stage, age, T stage, N stage and M stage. We believe that this prognostic model can improve the management of BLCA patients.

The XC system transfers cystine from the cell and converts it into cysteine for the synthesis of glutathione (GSH). The production and maintenance of GSH is essential to protect cells from oxidative stress. GSH scavenges lipid peroxidation to inhibit ferroptosis [31]. The prognostic risk score model proposed in this study was composed of 7 FRGs (X1). These genes were roughly divided into two categories: ACSL4, ALOX5, ACACA, ZEB1 and FADS2 are involved in lipid metabolism, and NOX1 and GCLM are involved in (anti)oxidant metabolism [9, 10].

ACSL4 is an important enzyme in pro-ferroptotic lipid metabolism. Knockdown of ACSL4 can reduce the ferroptosis of epithelial cells in lung ischaemia-reperfusion injury and can protect lung epithelial cells [32]. ALOX5 is an iron-containing nonheme dioxygenase that can limit leukotriene biosynthesis and catalyse the peroxidation of arachidonic acid, thereby mediating lipid peroxidation. ALOX5 controls cell ferroptosis by regulating cell inflammation and lipid peroxidation, suggesting that ALOX5 contributes to ferroptosis [33]. GCLM is the rate-limiting and first enzyme in the synthesis of glutathione, which is the main component of cellular oxidative stress. The deletion of its modified subunits can cause a significant decrease in glutathione levels in all body tissues, including the brain [34], so ferroptosis increases. ACACA is the first important step in catalysing the synthesis of fatty acids in the cytoplasm of mammals and is a key gene that regulates tumour cell survival. Knockout of ACACA can inhibit the ferroptosis induced by pharmacological agents [35]. ZEB1 plays an important role in cell lipid metabolism. Increasing evidence shows that ZEB1 can regulate lipid uptake, accumulation and mobilization and affect plasma membrane remodelling associated with EMT to change the process of cell ferroptosis [36]. Knockout of ZEB1 can inhibit the ferroptosis caused by GPX4 depletion [36]. FADS2 is related to many chronic diseases, including obesity, type 2 diabetes and metabolic abnormalities. FADS2 is essential for maintaining the body’s long-chain polyunsaturated fatty acid homeostasis. Knocking down FADS2 in lung cancer cells results in a significantly reduced cell growth rate, increased intracellular iron and lipid ROS levels and increased protein kinase-induced cell death [37, 38]. NOX1 is a type of NADPH oxidase that is the main source of reactive oxygen species (ROS) that regulate redox-sensitive signalling pathways. The use of NOX1 inhibitors can significantly reduce the ROS, lipid ROS and cell death of non-small cell lung cancer induced by erastin [39]. Because there are few studies on these genes, it remains to be clarified whether they play a role in the prognosis of BLCA patients by affecting the ferroptosis process.

By investigating the specific functions of the 7 FRGs, previous studies have shown that most of these genes play a key role in cancer cells, including BLCA. The abnormal expression of ZEB1 in BLCA is related to the differentiation and metastasis of bladder tumours. We used the Human Protein Atlas to verify the expression of ZEB1 in BLCA. ZEB1 was found to be highly expressed in BLCA tissue compared with normal bladder tissue. This protein can be used as a candidate target for prognosis prediction and early diagnosis [40]. In BLCA cells, knocking down NOX1 can reduce cell ROS production, leading to BLCA cell apoptosis [41]. Knockdown of ACSL4 expression can significantly attenuate the lipid peroxidation and ferroptosis induced by sorafenib in Huh7 cells and save the sorafenib-induced growth inhibition of xenograft tumours in vivo. ACSL4 is a key factor in sorafenib-induced ferroptosis and is useful for predicting the sensitivity of HCC to sorafenib [42]. Overexpression of ALOX5 in a PTC cell line (BCPAP) can increase cell invasion across the ECM barrier and the secretion of MMP-9. ALOX5 can be used as a new mediator of cell invasion induced by MMP-9 [43]. The mRNA and protein expression levels of FADS2 in human mesenteric tissues are reduced. The increased expression of FADS2 converts n-3 fatty acids into decomposable lipid mediators, resulting in a significant reduction in the infiltration of proinflammatory macrophages and weakened expression of inflammatory cytokines or adipokines. FADS2 may improve Crohn’s disease treatment [44]. The Human Protein Atlas database analysis showed that FADS2 expression is significantly higher in BLCA tissues than in normal bladder tissues. These results were consistent with previous bioinformatics analysis results (Fig. 2a, b). Therefore, the FRGs have different manifestations in diseases, and their specific roles need to be clarified.

Although the mechanism of ferroptosis has been the focus of research in the past, the potential relationship between ferroptosis and tumour immunity is still elusive. Our ssGSEA results showed that the scores of Macrophages, Mast_cells, pDCs, Tfh, Treg, APC_co_stimulation, CCR, Check-point and T_cell_co-inhibition were significantly higher in the high-risk group. These results suggest that ferroptosis may be closely related to tumour immunity. Studies have shown that an increase in tumour-related Treg cells [45] or macrophages [46] is related to the poor prognosis of HCC patients. In this study, the antigen presentation process was obviously different between the high-risk group and the low-risk group. One speculation is that ferroptotic cells may release lipid mediators to recruit antigen-presenting cells (APCs) to the location of ferroptotic cells [47]. In addition, a higher risk score was associated with decreased antitumour immunity, including the fractions of pDCs, Tfh and Mast_cells, and the activity of checkpoint and T_cell_co-inhibition. Therefore, the weakened antitumour immunity of high-risk patients may lead to poor prognosis. The CCK-8 proliferation assay showed that the proliferation ability of ZEB1- and FADS2-knockdown T24 cells was significantly reduced compared with that of control T24 cells. This finding suggests that ZEB1 and FADS2 may play an oncogenic role in BLCA, but the specific mechanism needs further study.

This study also has several limitations. First, our prognostic model was constructed with retrospective data from the TCGA database. Some cellular studies and animal experiments should be conducted on the 7 FRGs alone or in combination to check the predictive accuracy of the model and discover potential mechanisms. Second, it is necessary to use prospective real-world data to verify their clinical applicability. Third, using only a single hallmark to establish a prognostic model is flawed, as many important prognostic genes in BLCA may have been excluded. Finally, the links between immune status and the risk score have not yet been experimentally verified.

## Conclusion

In conclusion, we constructed a new prognostic model with 7 FRGs, which was proven to be independently related to OS and can accurately predict the prognosis of BLCA. Understanding the underlying mechanisms and significance of these FRGs in BLCA can provide insights for determining therapeutic targets of BLCA.

## Supplementary Information

**Additional file 1: Supplementary Table S1.** Sixty ferroptosis-related genes.

**Additional file 2: Supplementary Table S2.** Sixteen immune cells and thirteen immune-related pathways.

## Data Availability

All data generated or analyzed during the present study were downloaded from TCGA database (https://portal.gdc.cancer.gov/repository?facetTab=cases).

## Abbreviations

AUC: Area under the curve
BLCA: Bladder cancer
DEGs: Differentially expressed genes
FRGs: Ferroptosis-related genes
GO: Gene Ontology
KEGG: Kyoto Encyclopedia of Genes and Genomes
OS: Overall survival
PCA: Principal component analysis
ROC: Receiver operating characteristic
ssGSEA: Single-sample gene set enrichment analysis
TCGA: The Cancer Genome Atlas
