# Supplementary material for: A novel survival model based on a Ferroptosis-related gene signature for predicting overall survival in bladder cancer
Source: BMC Cancer. 2021 Aug 21;21:943. doi: 10.1186/s12885-021-08687-7 (PMC8380338; doi:10.1186/s12885-021-08687-7)
Supplement: Supplementary file 1 — Additional file 1: Supplementary Table S1. Sixty ferroptosis-related genes. [file 12885_2021_8687_MOESM1_ESM.docx]

**A Novel Survival Model Based on** **a Ferroptosis-related Gene Signature for Predicting Overall Survival in** **Bladder Cancer**

**Authors:**

Yingchun Liang^1,2,#^, Fangdie Ye^1,2,#^, Chenyang Xu^1,2^, Lujia Zou^1,2^, Yun Hu^1,2^, Jimeng Hu^1,2*^, Haowen Jiang^1,2,3*,^

^1^Departments of Urology, Huashan Hospital, Fudan University, No. 12 WuLuMuQi Middle Road, 200040 Shanghai, China.

^2^Fudan Institute of Urology, Huashan Hospital, Fudan University, Shanghai, China

^3^National Clinical Research Center for Aging and Medicine, Fudan University, Shanghai, China

^#^Yingchun Liang and Fangdie Ye contributed equally to this work.

***Corresponding Author:**

Jimeng Hu, E‑mail: jmhu14@fudan.edu.cn

Haowen Jiang, E‑mail: haowj_sh@fudan.edu.cn

**Supplementary Table S1 Sixty ferroptosis-related genes.**

| Ferroptosis-related genes | | | | | |
| --- | --- | --- | --- | --- | --- |
| ACSL4 | CARS1 | GCLC | LPCAT3 | TFRC | STEAP3 |
| AKR1C1 | CBS | GCLM | MT1G | TP53 | NFS1 |
| AKR1C2 | CD44 | GLS2 | NCOA4 | EMC2 | ACSL3 |
| AKR1C3 | CHAC1 | GPX4 | PTGS2 | AIFM2 | ACACA |
| ALOX15 | CISD1 | GSS | RPL8 | PHKG2 | PEBP1 |
| ALOX5 | CS | HMGCR | SAT1 | HSBP1 | ZEB1 |
| ALOX12 | DPP4 | HSPB1 | SLC7A11 | ACO1 | SQLE |
| ATP5MC3 | FANCD2 | CRYAB | FDFT1 | FTH1 | FADS2 |
| PGD | IREB2 | HMOX1 | ACSF2 | NOX1 | ABCC1 |
| NFE2L2 | KEAP1 | NQO1 | SLC1A5 | GOT1 | G6PD |
