# Supplementary material for: A novel survival model based on a Ferroptosis-related gene signature for predicting overall survival in bladder cancer
Source: BMC Cancer. 2021 Aug 21;21:943. doi: 10.1186/s12885-021-08687-7 (PMC8380338; doi:10.1186/s12885-021-08687-7)
Supplement: Supplementary file 2 — Additional file 2: Supplementary Table S2. Sixteen immune cells and thirteen immune-related pathways. [file 12885_2021_8687_MOESM2_ESM.docx]

**A Novel Survival Model Based on** **a Ferroptosis-related Gene Signature for Predicting Overall Survival in** **Bladder Cancer**

**Authors:**

Yingchun Liang^1,2,#^, Fangdie Ye^1,2,#^, Chenyang Xu^1,2^, Lujia Zou^1,2^, Yun Hu^1,2^, Jimeng Hu^1,2*^, Haowen Jiang^1,2,3*,^

^1^Departments of Urology, Huashan Hospital, Fudan University, No. 12 WuLuMuQi Middle Road, 200040 Shanghai, China.

^2^Fudan Institute of Urology, Huashan Hospital, Fudan University, Shanghai, China

^3^National Clinical Research Center for Aging and Medicine, Fudan University, Shanghai, China

^#^Yingchun Liang and Fangdie Ye contributed equally to this work.

***Corresponding Author:**

Jimeng Hu, E‑mail: jmhu14@fudan.edu.cn

Haowen Jiang, E‑mail: haowj_sh@fudan.edu.cn

**Supplementary Table S2** **Sixteen immune cells thirteen immune-related pathways.**

| immune cells | immune-related pathways |
| --- | --- |
| aDCs | APC_co_inhibition |
| B_cells | APC_co_stimulation |
| CD8+_T_cells | CCR |
| DCs | Check-point |
| iDCs | Cytolytic_activity |
| Macrophages | HLA |
| Mast_cells | Inflammation-promoting |
| Neutrophils | MHC_class_I |
| NK_cells | Parainflammation |
| pDCs | T_cell_co-inhibition |
| T_helper_cells | T_cell_co-stimulation |
| Tfh | Type_I_IFN_Reponse |
| Th1_cells | Type_II_IFN_Reponse |
| Th2_cells |  |
| TIL |  |
| Treg |  |
